# Occult hepatitis B virus infection among patients with chronic liver disease of unidentified cause, Addis Ababa Ethiopia

**DOI:** 10.1038/s41598-022-17336-3

**Published:** 2022-08-01

**Authors:** Selam Bogale Gissa, Mengistu Erkie Minaye, Biruk Yeshitela, Gizachew Gemechu, Abebech Tesfaye, Dawit Hailu Alemayehu, Abel Shewaye, Amir Sultan, Adane Mihret, Andargachew Mulu

**Affiliations:** 1 Department of Internal Medicine, Addis Ababa University, Addis Ababa, Ethiopia; 2 Department of Internal Medicine, Yekatit 12 Hospital Medical College, Addis Ababa, Ethiopia; 3 Armauer Hansen Research Institute (AHRI), Addis Ababa, Ethiopia; 4 ALERT Hospital, Addis Ababa, Ethiopia

**Keywords:** Diseases, Health care, Medical research

## Abstract

Occult hepatitis B virus infection (OBI) characterized by the absence of detectable HBsAg in the presence of HBV DNA in the serum and/or liver tissue remains a potential risk of transmission and diseases progression among different population groups. It could be associated with asymptomatic case up to chronic liver disease (CLD) and hepatocellular carcinoma (HCC). The objective of this study was to assess the magnitude and characteristics of OBI among patients with CLD of unidentified cause in Addis Ababa, Ethiopia. The study was conducted at the gastroenterology & hepatology referral clinic of three government and two private hospitals in Addis Ababa. Known CLD patients as evidenced by clinical and imaging criteria and/or with HBV surface antigen (HBsAg) negative results using rapid test kit were included. ELISA serological test to anti-HBc Ab, anti HBsAg Ab, and HBsAg were determined using BIORAD kits [https://www.bio-rad.com]. HBV-DNA was amplified, and viral loads were determined by quantitative real-time PCR using Abbott m2000rt platform following the manufacturer's instructions. Data analysis was done using SPSS version 20.A total of 48 CLD patients with no identified cause for their liver disease were identified during the study period. All the patients had evidence of CLD by clinical and imaging criteria and nine were excluded. Three (7.69%) of the 39 patients tested positive for HBsAg test done by ELISA making the negative predictive value of the rapid test kits 92.3% compared to ELISA. The remaining 36 patients had serology test for HBV and 16 (44.4%) had positive anti-HBV core antibody. Two (5.56%) of the 36 patients with HBV viral load determination had detectable HBV DNA suggesting presence of an occult hepatitis B infection. Occult hepatitis B infection is found to be an aetiology among CLD patients labelled as having no identified cause by the current standard of care using rapid HBsAg kits in a subset of patients in Ethiopia. This study signifies the high rate of OBI and past evidence of HBV infection among CLD patients and thus nucleic acid testing and/or anti-HBc shall be integrated to the routine health care system to minimize HBV infection risk of transmission and to enhance patient care.

## Introduction

Globally chronic liver disease (CLD) causes significant morbidity and mortality. In 2017 CLD was the 3rd and 10th leading cause of morbidity in males and females respectively with 1.3 million deaths in the same year. The majority of CLD worldwide is caused by Hepatitis B virus (HBV), hepatitis C virus (HCV), alcohol and non-alcoholic steatohepatitis (NASH) though the contribution of each shows marked geographic variability^1^. Sub-Saharan Africa has the highest death rate from cirrhosis in the world with a doubling of cirrhosis-related death between 1980 and 2010. Young and middle-aged adults are especially more affected. Despite the availability of effective treatment viral hepatitis remain the commonest causes of CLD and cirrhosis in Africa. In 2017 in eastern sub-Saharan Africa HCV and HBV were responsible for 29% and 26% of CLD and cirrhosis cases respectively^2,3^.

In Sub-Saharan Africa estimates of the prevalence of HBV range from 0.5 to 20%; however, HBV surface antigen (HBsAg) prevalence of as much as 38% have been reported^4–6^. The spectrum of HBV related disease is broad and includes acute hepatitis (which may progress to fulminant hepatic failure), an asymptomatic carrier state, an immune-tolerant chronic carrier state, and patients with active inflammatory disease characterized by the presence of a high viral load and high serum transaminases. The hallmark of all these states is the presence of HBsAg, which is detectable via blood sampling^6^.

In addition to overt HBV infection, there is an occult hepatitis B infection (OBI) which is characterized by the absence of HBV surface antigen (which is the commonest initial diagnostic test for HBV diagnosis) and the presence of HBV DNA in the serum or liver with or without immunologic evidence of HBV infection. OBI has been increasingly recognized as causing CLD, hepatocellular carcinoma, reactivation with the use of certain drugs (e.g., immunosuppressants and direct acting oral antiviral for HCV) and potential transfer of HBV by blood donors who are only screened by HBV surface antigen. Debate continues about the reason why Hepatitis B surface antigen will be negative in the presence of viral DNA and its full clinical implications. Various mechanisms including change in the viral genome, host immune, co- infections and quality of test have been proposed by various authors on the genesis of OBI^7–13^.

Ethiopia has a high burden of CLD and is endemic for HBV with a reported pooled prevalence 9.4%^14^. Initial diagnostic test for HBV in Ethiopia is mostly done using rapid test kits for HBV surface antigen. Few studies that attempted to identify causes of CLD have shown a variable proportion (ranging from 14 to 55%) of CLD patients remain having no identified cause for the liver disease. In previous studies from the country the proportion of unexplained CLD is very high approaching 50% of all patients with cirrhosis in some localities^15,16^. There are few studies on OBI in the Ethiopian setting. Two studies conducted in east and north Ethiopia had shown OBI prevalence to be around 6% and 19%, respectively among HBV core antibody positive clients. Most of the participants in these two studies were HIV positive and on HAART, with no evidence of decompensated liver disease^17,18^. Another study from north Ethiopia has shown OBI to occur in nearly 20% of pregnant mothers attending maternity clinic and were anti HBV core antibody positive^19^.

However, to the authors knowledge there is no published report regarding the magnitude and impact of OBI among patients with CLD of unidentified cause in Ethiopian context. This study is therefore, intended to determine the magnitude, serological patterns, virologic and clinical characteristics of OBI among patients with unidentified causes of chronic liver disease in Addis Ababa, Ethiopia.

## Methods

### Study design

A cross-sectional study was conducted among patients with CLD of unidentified cause who presented between September 2020 and January 2021 at gastroenterology/ hepatology referral units of 3 government hospitals (Tikur Anbassa Specialized Hospital, Yekatit 12 Hospital Medical College and Armed Force Referral and Teaching Hospital) and 2 private health facilities (Hallelujah Hospital and Yehulshet Clinic) in Addis Ababa, Ethiopia. The age of patients was 13 and above and all of them had clinical and imaging evidence of CLD, all of them diagnosed at least six months before. These patients have no identifiable cause for the CLD based on existing diagnostic tools and are only under supportive treatments. Those with known causes of liver disease and/ or taking specific treatment(s) were excluded from the study. Furthermore, patients with HBV/HCV infection, significant alcohol intake, drug/ toxin induced liver disease, heart failure, hepatosplenic schistosomiasis, diagnosed vascular, autoimmune causes, those taking immunosuppressants, were HIV positive and on HAART were also excluded. HBV infection was ruled out using rapid test kit for HBV surface antigen (ACON: ACON Laboratories. San Diego, CA, USA, and Advanced Quality (InTec Products Inc. Anaheim, CA, USA) at their respective care centers; each patient had either of the two rapid tests but not both. The lower limit of detection (LoD) of ACON was 0.79 ng/ml for detecting HBV surface antigen, while LoD of the later was not mentioned in the kit’s leaflet.

### Data collection and laboratory tests

Sociodemographic and clinical variables were captured during the routine clinical examination of patents. A further medical record review via structured questionnaire was made. Five ml of venous blood was also taken; plasma was separated and stored at -80° C until further investigated. Evidence of HBV infection was again assessed using HBsAg (ELISA Monolisa, Biorad, France), Anti HBc and Anti HBsAg Antibody tests (ELISA Monolisa, Biorad, France) and quantitative real-time PCR using Abbott m2000rt platform (Abbott, Germany) following the manufacturer's instructions. The lowest limit of HBV viral load of the PCR was 15 IU/ml. The LoD of ELISA Monolisa test for HBsAg was 0.025 IU/ml.Test results for serology and HBV DNA were interpreted according to the manufacturer’s protocol. All methods and laboratory procedures were performed as per relevant guidelines and regulations as well as manufacturers' instructions.

### Statistical analysis

Data were checked for completeness and entered and analyzed using SPSS v20 (SPSS Inc., Chicago, IL, USA). Categorical variables were summarized using frequencies and percentages. Continuous variables were presented using mean, standard deviation, and range.

### Ethics

The research protocol was approved by the Department Research Ethics Committee (DREC) of Department of Internal Medicine, School of Medicine, Addis Ababa University (Meeting number: 017/20, Protocol number: 043/20). Participants older that 18 years gave an informed written consent and for those below age 18 years an informed written consent was obtained from their guardians. All principles of research ethics were maintained during the study.

## Results

A total of 48 patients with the diagnosis of CLD who were seen during the study period were found to be eligible. Three refused to participate and 6 were excluded for having incomplete data and/or presence of exclusion criteria on detailed review of their medical records. Finally, a total of 39 patients with CLD of unidentified cause at the clinical site were included in the study.

Three of the 39 patients were found to have positive ELISA for HBsAg and excluded from the final test for OBI. The remaining 36 participants with negative HBsAg were further investigated for OBI. Most (55.6%) of the study participants were between the ages of 18–39 years with mean age of 35.78 (± SD = 16.937) year (Table 1).

**Table 1:** Sociodemographic characteristics of patients with chronic liver disease of unidentified cause (n = 36).

Variable	Category	Frequency (%)
Age category (years)	13–17	4 (11.1)
	18–39	20 (55.6)
	≥ 40	12 (33.3)
Sex	Male	20 (55.6)
	Female	16 (44.4)
Educational status	Unable to read and write	3 (8.3)
	Able to read and write only	2 (5.6)
	Primary (1–8)	13 (36.1)
	Secondary (9–12)	8 (22.2)
	Technical/vocational	3 (8.3)
	University	7 (19.4)
Marital status	Single	14 (38.9)
	Married	20 (55.6)
	Widowed	2 (5.6)
Residence	Urban	27 (75. 0)
	Rural	9 (25. 0)
Number of other households’ occupants	None	2 (5.6)
	1–4	21 (58.3)
	≥ 5	13 (36.1)
Patient care setting	Government	31 (86.1)
	Private	5 (13.9)

Twenty-seven (75%) patients had upper gastrointestinal (GI)—bleeding and/ or ascites while 9(25%) had neither. Twenty-two patients have upper gastrointestinal endoscopy of whom 16 have varices. Tables 2, 3, and supplement 1 show the clinical, laboratory and imaging characteristics of the study participants.

**Table 2:** Risk factors and clinical characteristics for HBV of patients with chronic liver disease of unidentified cause and their clinical characteristics(n = 36).

Variable	Category	Frequency (%)
History of tattoo including gum	Yes	6 (16.7)
	No	30 (83.3)
History of more than one lifetime sexual partner	Yes	8 (22.2)
	No	28 (77.8)
History of blood transfusion after the current illness started	Yes	7 (19.4)
	No	29 (80.6)
History of blood transfusion before developing the current disease	Yes	2 (5.6)
	No	34 (94.4)
Family history of liver disease (without known cause)	Yes	3 (8.3)
	No	33 (91.7)
Ascites	Yes	18 (50.0)
	No	18 (50.0)
Upper gastrointestinal bleeding	Yes	14 (38.9)
	No	22 (61.1)
Current use of diuretics	Yes	15 (41.7)
	No	21 (58.3)
Current use of beta blocker	Yes	23 (63.9)
	No	13 (36.1)

**Table 3:** Imaging results of patients with chronic liver disease of unidentified cause(n = 36).

Imaging	Index	Frequency (%)
Ultrasound	Cirrhosis	26 (72.2%)
	Hepatocellular carcinoma	2 (5.6%)
	Cirrhosis + hepatocellular carcinoma	2 (5.6%)
	Enlarged and bright liver	1 (2.8%)
	Hepato-splenomegaly with portal hypertension	1 (2.8%)
	Normal	1 (2.8%)
	No report	3 (8.3%)
CT scan	Cirrhosis	4 (11.1%)
	Hepatocellular carcinoma	2 (5.6%)
	Cirrhosis + hepatocellular carcinoma	1 (2.8%)
	Enlarged and bright liver	1 (2.8%)
	Hepato-splenomegaly with portal hypertension	1 (2.8%)
	No report	27 (75%)

The 36 patients with negative test for HBsAg by ELISA were further tested for evidence of OBI. HBV serology has shown that 17 (47.2%) were positive for anti HBV core antibody. There were 11 (30.6%) and one (2.8%) serology result with immunity from past infection and vaccination respectively, while 6 (16.7% patients have isolated anti HBV core antibody (Supplement 2). HBV viral load result was available for 36 of the study participants. Two (5.56%) of the 36 participants had detectable HBV DNA with HBV viral load values of 45 and 643 IU/ml. Both patients with OBI have detectable anti core antibody while only one of the two was positive for Ant HBs antibody.

The first patient with OBI had viral load of 45 IU/ml had CT scan suggesting HCC with cirrhosis and ascites and the second OBI patients had ultrasound reading suggestive of cirrhosis and has ascites with viral load of 643 IU/ml. Both are male from urban area with the ages of 75 and 45 years old, respectively. Among the three patients with negative result for HBsAg by rapid kit but positive by ELISA two had detectable viral loads of 25 and 33 IU/ml.

## Discussion

Occult hepatitis B virus infection is one possible cause of CLD in HBV endemic areas. This study describes for the first time the prevalence and characteristics of OBI among well characterized patients with CLD of unidentified cause from African settings of Addis Ababa, Ethiopia.The study found that close to half of CLD patients with no identified cause have serologic evidence of past HBV infection among. Though it was not our primary objective and our sample size was small we found rapid tests for HBsAg to have lower negative predicative value compared to ELISA. All the 39 patients that fulfilled eligibility criteria had negative HBsAg done by 2 commonly available rapid tests. Three of this had a positive test for HBsAg by ELISA. Compared to ELISA (Monolisa)—the RDT (ACON), which was used for 31 (86.1%) of the study participants, has a NPV of 92.3% which is lower than NPV of 95% in a study done in Pakistan comparing this RDT with ELISA^20^. This NPV for the ACON RDT is also lower than a 99.2% NPV compared to ELISA in a study done in Iraq^21^. A similar kit had a NPV of 76.9% in a study from Togo which is lower than this study^22^. The above study shows variability of accuracy of RDT for HBsAg. Meta-analysis showed that different kits have different accuracy with reasonable specificity and sensitivity compared with ELISA and are cost effective approaches in population screening in endemic areas with limited resources^23,24^. In clinical practice however detecting 3 false negatives of 39 (7.6% of the participants) is high considering the impact it will have on the management of patients and hence affects prognosis.

In this study two of the 36 participants who were tested for HBV viral load had detectable HBV virus making OBI to be 5.56%. Both patients have imaging evidence of cirrhosis and ascites with one of them having HCC on background cirrhosis. Reports from Iran showed the prevalence of OBI among cryptogenic cirrhosis patients to be 2%, 14% and 38%^25–27^. While a study from Italy showed 12% and 20% in patients with cirrhosis of unknown cause^28,29^. While studies from China showed a 28.3% and 73% OBI among patients with cryptogenic CLD and 70.4% among HCC patients with no cause^30,31^. A study from Egypt showed 22.5% and 62.5% OBI when done from serum & liver tissue, respectively^32^. Another study showed OBI to be present in 73% of HCC patients from Japan^33^. While a study in South Africa showed 48% prevalence of OBI in HCC patients negative for HBsAg^34^.

The above studies show a wide range of prevalence of OBI among cirrhosis and HCC of unidentified cause. Occult hepatitis B infection among patients vary based on whether HBV viral load is done from serum or liver tissue. As with HBV prevalence that of OBI could also have geospatial variation. Other factors that contribute to the variability include the lower limit of detection of the PCR method used and fluctuation of HBV DNA levels. And hence, OBI prevalent studies should be interpreted carefully. Mechanisms implicated in the development of OBI include viral factors (genetic and epigenetic), host immune factors and co-infection with other infectious agents^12^.

Fifty percent of the liver disease patients in this study had HBV serology profile of being susceptible for HBV infection which is considerably high for patients in moderate to high endemic area with established liver disease. This may potentially render patients for more illness and decompensation. Seventeen (47.23%) patients had anti core antibody. The viral load in such patients might fluctuate requiring serial measurements as a higher number of patients might have an occult infection.

This study is the first of its kind in the Ethiopian setting to look for OBI among CLD patients of unidentified cause. We have tested both serology markers and viral load for OBI and most of the common causes of CLD in Ethiopia were excluded. However, the study has obvious limitations including small sample size, absence of all HBV serological markers and detailed investigation for other causes liver disease. Furthermore, use of one-time plasma alone instead of using both plasma and liver tissue to diagnose OBI might underestimate the true burden of OBI in the settings. ELISA for the antibodies were also done using one test type due to resource.

## Conclusion

This study has shown that significant proportion of CLD patients with no identified cause had evidence of past and/or occult hepatitis B infection. Rapid test for hepatitis B might also miss this group of patients. We strongly recommend further multicentric prospective study with interventions to contribute to building solid data that will help to guide future testing and treatment recommendations of occult hepatitis B infection among CLD patients with no identified cause. Thus, nucleic acid testing and/or anti-HBc shall be integrated to the routine health care system to minimize the HBV infection risk of transmission and to enhance patient care.

## Supplementary Information

Supplementary Information 1.

Supplementary Information 2.

## Data Availability

All data pertaining the results of this study are included in the manuscript and supplement.
