# Supplementary material for: Occult hepatitis B virus infection among patients with chronic liver disease of unidentified cause, Addis Ababa Ethiopia
Source: Sci Rep. 2022 Aug 1;12:13188. doi: 10.1038/s41598-022-17336-3 (PMC9343390; doi:10.1038/s41598-022-17336-3)
Supplement: Supplementary file 1 — Supplementary Information 1. [file 41598_2022_17336_MOESM1_ESM.pdf]

Variable	Mean (±SD)	Range
Platelet count (n=35) thousand per micL	119.89 (100.33)	22.00-474.00
Hemoglobin (n=34) in g/dl	12.79 (2.19)	7.80 - 17.40
WBC (n=34) in thousand per micL	4.13 (2.01)	1.20 - 17.00
Albumin (n=36) in g/dl	3.31 (0.98)	1.31 - 4.80
AST (n=36) in IU/L	52.61 (35.42)	15.00 – 164.00
ALT (n=36) in IU/L	35.14 (25.22)	10.00 – 132.00
Alkaline phosphatase(n=33) in IU/L	169.24 (96.78)	39.00 – 463.00
Total bilirubin(n=36) in mg/d	1.84 (1.835)	0.30 - 7.80
direct bilirubin (n=36) in mg/dl	0.78 (1.126)	0.01 - 4.62
Urea (n=33) in mg/dl	18.94 (7.22)	6.00 - 47.00
Creatinine (n= 35) in mg/dl	0.64 (0.26)	0.20 - 1.50

Supplement 1. Basic laboratory profile of patients with chronic liver disease of unidentified cause(n=36)
