# Supplementary material for: Occult hepatitis B virus infection among patients with chronic liver disease of unidentified cause, Addis Ababa Ethiopia
Source: Sci Rep. 2022 Aug 1;12:13188. doi: 10.1038/s41598-022-17336-3 (PMC9343390; doi:10.1038/s41598-022-17336-3)
Supplement: Supplementary file 2 — Supplementary Information 2. [file 41598_2022_17336_MOESM2_ESM.pdf]

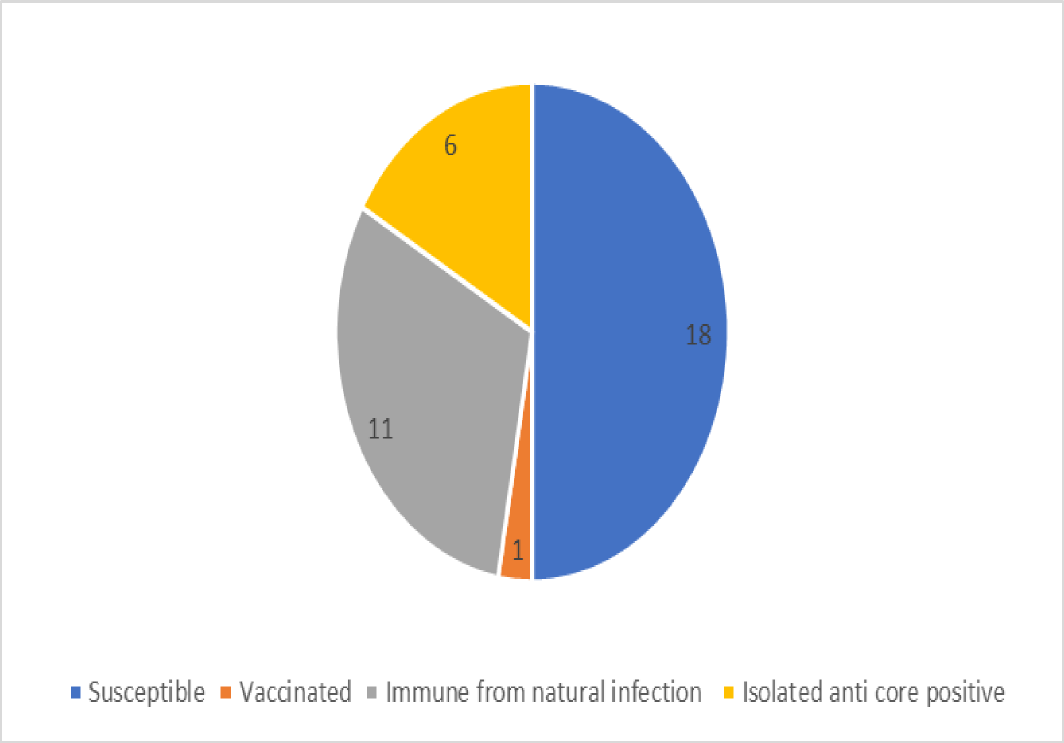

Supplement 2: Frequency of HBV serology interpretation of patients with CLD & HCC of unidentified cause in Addis Ababa, Ethiopia. March 2021

*susceptible= Anti HBcAg Antibody and Anti HBsAg antibody negative

*Vaccinated= Only Anti HBsAg antibody positive

* Immune from natural infection= Anti HBcAg Antibody and Anti HBsAg antibody positive

* Isolated anti core positive = Only Anti HBcAg Antibody positive
